# Damage control resuscitation: a practical approach for severely hemorrhagic patients and its effects on trauma surgery

**DOI:** 10.1186/s40560-016-0197-5

**Published:** 2017-01-20

**Authors:** Yasumitsu Mizobata

**Affiliations:** Department of Traumatology and Critical Care Medicine, Graduate School of Medicine, Osaka City University, 1-4-3 Asahimachi, Abeno-ku, Osaka City, Osaka 545-8585 Japan

**Keywords:** Damage control resuscitation, Acute traumatic coagulopathy, Massive transfusion protocol, Damage control surgery, Balanced resuscitation

## Abstract

Coagulopathy observed in trauma patients was thought to be a resuscitation-associated phenomenon. The replacement of lost and consumed coagulation factors was the mainstay in the resuscitation of hemorrhagic shock for many decades. Twenty years ago, damage control surgery (DCS) was implemented to challenge the coagulopathy of trauma. It consists of three steps: abbreviated surgery to control the hemorrhage and contamination, resuscitation in the intensive care unit (ICU), and planned re-operation with definitive surgery. The resuscitation strategy of DCS focused on the rapid reversal of acidosis and prevention of hypothermia through the first two steps. However, direct treatment of coagulopathy was not emphasized in DCS.

Recently, better understanding of the pathophysiology of coagulopathy in trauma patients has led to the logical opinion that we should directly address this coagulopathy during major trauma resuscitation. Damage control resuscitation (DCR), the strategic approach to the trauma patient who presents in extremis, consists of balanced resuscitation, hemostatic resuscitation, and prevention of acidosis, hypothermia, and hypocalcemia. In balanced resuscitation, fluid administration is restricted and hypotension is allowed until definitive hemostatic measures begin. The administration of blood products consisting of fresh frozen plasma, packed red blood cells, and platelets, the ratio of which resembles whole blood, is recommended early in the resuscitation.

DCR strategy is now the most beneficial measure available to address trauma-induced coagulopathy, and it can change the treatment strategy of trauma patients. DCS is now incorporated as a component of DCR. DCR as a structured intervention begins immediately after rapid initial assessment in the emergency room and progresses through the operating theater into the ICU in combination with DCS. By starting from ground zero with the performance of DCS, DCR allows the trauma surgeon to correct the coagulopathy of trauma. The effect of the reversal of coagulopathy in massively hemorrhagic patients may change the operative strategy with DCS.

## Background

Massive bleeding following injury remains the main cause of death in trauma patients. Uncontrolled hemorrhage is reported to be responsible for 40% of trauma deaths [1]. The central measure for controlling such bleeding incorporated physical hemostatic approaches, such as surgery or interventional radiology. Coagulopathy had been thought to be a resuscitation-induced phenomenon, and replacement of the lost and consumed coagulation factors was the mainstay in the resuscitation of hemorrhagic shock. Recently, better understanding of the pathophysiology of coagulopathy in trauma patients has led to the logical opinion that we should directly address coagulopathy during major trauma resuscitation. Damage control resuscitation (DCR) is a strategic approach to the trauma patient who presents in extremis. In this review article, the pathophysiology of the coagulopathy in trauma patients, the theoretical and practical aspects of DCR, and the revolution of damage control surgery (DCS) incorporated with DCR are discussed.

## Coagulopathy in trauma

### Resuscitation-associated coagulopathy

Traditionally, the coagulopathy observed in trauma patients was thought to be “resuscitation-associated coagulopathy,” which is caused by the consumption of coagulation factors, dilution of coagulation factors after massive infusion, hypothermia, and acidosis. An increasing incidence of coagulopathy was observed with increasing amounts of intravenous fluids administered [2]. The administration of large amounts of fluids and blood products, exposure of the body, and surgical intervention performed for resuscitation cause the hypothermia. The alcohol and drugs, which are one of the causes of trauma incident, increase heat loss from the trauma patient. Hypothermia is observed in about 60% of trauma patients who require emergency operative interventions [3]. It is associated with platelet dysfunction and reduced enzyme activities [4] and an increased risk of bleeding and mortality of trauma patients [5]. Inadequate tissue perfusion due to hemorrhagic shock results in anaerobic metabolism and the subsequent production of lactic acid, which causes metabolic acidosis. The high chloride content in crystalloid solutions, such as 0.9% normal saline, exacerbates the metabolic acidosis [6, 7]. The activity of most of the coagulation factors is dependent on the blood pH. For example, the activity of factors VIIa and Xa/Va decreases by over 90% [8] and 70% [9], respectively, when the blood pH decreases from 7.4 to 7.0.

### A vicious cycle

In 1982, Kashuk and his colleagues emphasized the importance of coagulopathy in their clinical review of 161 patients with major abdominal vascular injury [10]. They reported that most of the deaths were a result of hemorrhage, and overt coagulopathy was identified in 51% of patients after vascular control was achieved.

The term “lethal triad” was used to describe the physiologic derangement observed in these patients and refers to the triad of the deteriorating status of acute coagulopathy, hypothermia, and acidosis of exsanguinating trauma patients. The lethal triad forms a downward spiral, and further hemorrhage deteriorates the triad. Unless this cycle can be broken, the patient’s death is unavoidable. From this aspect, this downward spiral is known as the “vicious cycle of trauma” or the “bloody vicious cycle,” which demands as much attention from the physician as the classically emphasized initial resuscitation and operative intervention.

### Acute traumatic coagulopathy

Recently, injury itself is reported to cause early coagulopathy [11, 12], which is known as “trauma-induced coagulopathy” [13] or “acute traumatic coagulopathy (ATC)” [14]. ATC is an obvious early coagulopathy and occurs prior to significant dilution [14, 15], within 30 min of injury [12], and affects a quarter of the patients with severe trauma [14]. The patients with this coagulopathy have higher mortality than those with normal clotting function [14].

Although the pathophysiology of ATC is not fully understood, it is thought to occur following injury and concomitant hypoperfusion [16]. ATC is affected primarily through activated protein C, which causes both anticoagulant effects and fibrinolytic effects by inhibiting plasminogen activator inhibitor-1. Instead of the importance of tissue factor, another group has argued that the coagulopathy in trauma is one of disseminated intravascular coagulation with a fibrinolytic phenotype, which is characterized by activation of the coagulation pathways, insufficient anticoagulation mechanisms, and increased fibrinolysis [17, 18].

These recent understandings of ATC have guided the principle and practice of DCR, which directly addresses the hemostatic dysfunction of the severely injured patient.

## Damage control resuscitation

### Management of coagulopathy in trauma patients

In the severely injured patient, unless the lethal triad of hypothermia, acidosis, and coagulopathy is prevented, death is unavoidable [19]. DCS is a resuscitation strategy that was devised to avoid these physiological disorders. It consists of three steps: abbreviated surgery to control the hemorrhage and contamination, resuscitation in the intensive care unit (ICU), and planned re-operation with definitive surgery [20]. The resuscitation strategy of DCS focused on the rapid reversal of acidosis and the prevention of hypothermia through the first two steps. However, direct treatment of coagulopathy was not emphasized in DCS. The coagulopathy observed in hemorrhagic patients was thought to be a result of resuscitation, acidosis, and hypothermia. Thus, the aim of DCS was to avoid the acidosis and hypothermia resulting from aggressive definitive surgery. Little attention was paid to the early derangement of coagulation function caused by the trauma itself. In contrast, DCR directly addresses the trauma-induced coagulopathy immediately upon patient admission [21] or in the pre-hospital setting [22]. DCR consists of balanced resuscitation, hemostatic resuscitation, and prevention of acidosis, hypothermia, and hypocalcemia.

### Balanced resuscitation

The patient’s response to the rapid infusion of isotonic fluid or blood is the indicator of the need for surgical or interventional hemostatic procedures. Aggressive fluid resuscitation was the initial fluid therapy recommended for many decades. However, this approach may contribute to increased blood loss and higher mortality [23]. The warning concerning the massive administration of fluid was already reported about 100 years ago by Captain *Cannon* [24]. He commented that, “There is no doubt that in some cases such injections have had definitely beneficial effects, however, the injection of a fluid, that will increase blood pressure, has dangers in itself. If the pressure is raised before the surgeon is ready to check any bleeding, blood needed may be lost.”

Increasing evidence has shown that aggressive crystalloid-based resuscitation strategies are associated with cardiac and pulmonary complications [25], gastrointestinal dysfunction, coagulation disturbances, and disorders of immunological and inflammatory mediators [26]. The administration of large volumes of fluids results in imbalances of intracellular and extracellular osmolarity that affect cell volume. Disturbances in cell volume then disrupt numerous regulatory mechanisms responsible for controlling the inflammatory cascade.

For these reasons, an alternative approach to the treatment of hemorrhagic patients was recently proposed and practiced. The approach was introduced as permissive hypotension, delayed resuscitation, or controlled resuscitation. The aim of these resuscitation strategies is not hypotension but rather to balance the risk of decreased tissue perfusion with the benefits from the prevention of coagulopathy.

In 1994, Bickell and colleagues investigated the benefit of delayed fluid resuscitation in a randomized controlled trial. Five hundred eighty-nine adult patients with penetrating injuries and a pre-hospital systolic blood pressure of less than 90 mmHg were enrolled in the trial [27]. The application of delayed fluid resuscitation increased the survival rate of of the patients from 62 to 70%.

After this report, several randomized or retrospective studies concerning balanced resuscitation were reported; however, the benefit to mortality varied among the studies [28–31]. Turner et al. randomized more than 1000 patients to immediate or delayed resuscitation in the pre-hospital setting but showed no beneficial effects on mortality [28]. Both Dutton et al. and Morrison et al. investigated the effects of hypotensive resuscitation in about 100 patients, but the results varied between these two reports [29, 30]. Duke et al. retrospectively compared cohorts with standard and restricted fluid resuscitation and reported that restricted fluid resuscitation showed a survival benefit [31].

When evaluating the effects of balanced resuscitation, these results should be interpreted cautiously. The patients enrolled in the Bickell et al. and Duke et al. reports were victims of penetrating injury only. In the reports of Morrison et al. and Dutton et al., the rates of patients with penetrating injury were 93 and 51%, respectively. The time from hospital arrival to the emergency operation was very short, and furthermore, the patients were in their 20s or 30s. There are other concerns, such as the low protocol compliance in the Turner et al. report and the difficulty of controlling the blood pressure at the aimed-for level in the Dutton et al. and Morrison et al. reports.

The ninth edition of the Advanced Trauma Life Support emphasizes the concept of balanced resuscitation, and the term “aggressive resuscitation” has been eliminated. The standard use of 2 L of crystalloid resuscitation as the starting point for all resuscitation has been modified to the initiation of 1 L of crystalloid infusion. Early use of blood and blood products for patients in shock is emphasized [32].

The most recent randomized controlled trial to evaluate the efficacy of balanced resuscitation was reported in 2015 [33]. This multicenter study was performed in 19 emergency medical services systems in the USA and Canada. The controlled resuscitation resulted in a reduction of early crystalloid resuscitation volume and an increase in the early transfusion of blood products. Although mortality at 24 h was not different among all patients, it improved in the subgroup with blunt trauma. The controlled resuscitation strategy can be successfully and safely implemented in a civilian environment beginning with the out-of-hospital setting and extending into early hospital care.

### Hemostatic resuscitation

In 2007, Borgman and Holcomb et al. reported a survival benefit for the high ratio of plasma to red blood cell (RBC) in patients who received massive transfusions at a combat support hospital [34]. A high plasma to RBC ratio (1:1.4) was independently associated with improved survival, primarily by decreasing death from hemorrhage. Following this article, several studies investigating the survival benefit of a high ratio of fresh frozen plasma (FFP) to RBC were reported [35–40]. Although the ratio of FFP to RBC differed between the studies, a significant decrease in the mortality of the massively transfused patients in the high-ratio population as compared to the low-ratio population was achieved in both the civilian setting and the combat situation.

However, it remains controversial which ratio, 1:1 or 1:2, is beneficial and when the ratio should be achieved. Snyder et al. worried about the survival bias in the beneficial results observed in the retrospective studies [41]. Holcomb and colleagues investigated the relationship between in-hospital mortality and the early transfusion of plasma or platelets, and time variance in the delivery of plasma to RBC or platelet to RBC ratios in a multicenter prospective observational study [42]. The number of patients receiving the higher ratio rose as time passed. In the first 6 h, patients receiving a ratio of less than 1:2 were three to four times more likely to die than patients receiving a ratio of 1:1 or higher. They concluded that the earlier and higher ratio of plasma to RBCs decreased in-hospital mortality, and this beneficial effect was enhanced when the high ratio was achieved in the first 6 h after admission. In the Japan-Observational study for Coagulation and Thrombolysis in Early Trauma (J-OCTET), 189 severe trauma adult patients were registered [43]. Although the area under the curve was not high, the receiver operating characteristic curve analysis showed that the FFP/RBC ratio of 1.0 resulted in maximum sensitivity and specificity for survival. They concluded that a transfusion with an FFP/RBC ratio over 1.0 within the first 6 h reduces the risk of death by about 60% in patients with blunt hemorrhagic trauma.

The most recent randomized trial to evaluate the suitable ratio of plasma to RBCs for patients with severe trauma and major bleeding was performed in the pragmatic, randomized optimal platelet and plasma ratios (PROPPR) study [44], in which 680 patients were randomized to receive either a 1:1:1 or 1:1:2 ratio of plasma, platelets, and RBCs. Although the mortality was not significantly different between the two groups, more patients in the 1:1:1 group achieved hemostasis. Exsanguination, which was the predominant cause of death within the first 24 h, was significantly decreased in the high-ratio group.

### Rewarming

In DCR, hypothermia should be managed in conjunction with the efforts to correct the trauma-induced coagulopathy. It is essential to rewarm the torso using passive warming measures, such as insulting foil, blankets, and the removal of wet clothes. The initial fluid resuscitation should be carried out with warmed infusions at a fluid temperature of 40–42 °C [5, 45]. Heated air inhalation, gastric or body cavity lavage with warmed fluids, and heat radiation are widely performed as well as the standardized use of warming measures with rapid infusers. The temperature in the emergency room and the operating room should be raised, at best to a thermally neutral range (28–29 °C) [46]. If the hypothermia persists or quickly relapses despite these maximal rewarming efforts, ongoing hemorrhage and unresolved tissue hypoperfusion and hypoxia should be suspected.

### Reversing acidosis

Buffering of metabolic acidosis using drugs not only aggravates the intracellular acidosis but also does not reverse the coagulopathy [47]. Reversal of metabolic acidosis in the trauma patient is better obtained through fluid and blood resuscitation and vasopressor support with surgical control of hemorrhage. Shock should be reversed and end-organ perfusion is restored [48]. Because vital signs such as blood pressure and heart rate are not adequate to evaluate peripheral tissue perfusion, several endpoints of resuscitation are addressed. Base deficit and lactate levels are the reliable indices with which to evaluate the adequacy of the resuscitation and end-organ perfusion. Not only the initial lactate value upon admission but also lactate clearance from plasma within the first few hours of resuscitation correlate with the mortality of trauma patients [49, 50].

### Tranexamic acid

Because hyperfibrinolysis was recognized to contribute to the acute coagulopathy in trauma, administration of antifibrinolytic agents had theoretical benefit. The clinical randomization of an antifibrinolytic in significant hemorrhage 2 (CRASH-2) study, a large multi-center randomized controlled trial, investigated the effect of tranexamic acid on mortality and blood product requirements in trauma patients with hemorrhagic shock [51]. The study was undertaken in 274 hospitals in 40 countries. More than 20,000 adult trauma patients were randomized to receive either tranexamic acid or placebo within 8 h of injury. All-cause mortality and the risk of death due to bleeding were significantly decreased with the administration of tranexamic acid. Maximal beneficial effects were achieved if it was given within the first 3 h of injury. However, a recent study indicated that the majority of severely injured patients have fibrinolysis shutdown, and therefore, tranexamic acid may have no effect [52, 53]. The greatest benefit of tranexamic acid may be in patients in whom increased clot lysis is shown to be present using thromboelastography.

### Fibrinogen concentrates

Fibrinogen plays a central role in the coagulation process. It bridges activated platelets and works as the key substrate of thrombin to generate a stable fibrin mesh. In patients with blood loss, fibrinogen has been reported to decrease more rapidly under critically low concentrations than the other coagulation factors [54]. Thus, the supplementation of fibrinogen is a measure that makes sense when treating the coagulopathy of trauma patients. The effect of the administration of fibrinogen concentrates on outcome was investigated by matched-pairs analysis using the German Trauma Registry [55]. Although 30-day mortality was comparable, 6-h mortality was significantly lower in the patients receiving fibrinogen. The fibrinogen concentrates might have delayed the cause of death from early hemorrhagic collapse to late multiple organ failure.

### Prothrombin complex concentrate

Recently, prothrombin complex concentrate, derived from human plasma and contains variable amounts of factors II, VII, IX, and X, is used to correct coagulopathy [56, 57]. Goal-directed coagulation management using thromboelastometry was used to evaluate requirements of clotting factors [56, 57]. The administration of fibrinogen concentrate alone or in combination with prothrombin complex concentrate resulted in a significant improvement of fibrin polymerization and shorter clotting time [56]. Schochl et al. used fibrinogen concentrate and prothrombin concentrate complex as first-line therapies for coagulopathy based on thromboelastography in a study of 131 severely injured patients [57]. Transfusion of fresh frozen plasma and cryoprecipitate was avoided in the vast majority of these patients and outcomes were better than predicted.

### Cryoprecipitate

In the countries, where administration of fibrinogen concentrates is not approved in trauma patients, cryoprecipitate is the alternative treatment option as a source of fibrinogen. However, there are no reports suggesting positive effects of cryoprecipitate administration on the survival of exsanguinating trauma patients [58–60]. Although cryoprecipitate contains high concentrations of fibrinogen, it is hampered by several relevant disadvantages in terms of its availability, allogenicity, and the need for blood type matching and time-consuming thawing. Because the timing and indications for the administration of cryoprecipitate were unclear in the previously reported studies, a prospective randomized trial will be required to evaluate its benefit [59].

### Calcium

Calcium acts as an important cofactor in the coagulation cascade. Low levels of ionized calcium at admission are associated with increased mortality and an increased requirement for massive transfusion [61, 62]. Citrate, which is used as an anticoagulant in blood product components, chelates calcium and exacerbates the hypocalcemia, particularly when used in the FFP. The faster the transfusion is given, the faster the reduction of the calcium concentration occurs [63]. An ionized calcium concentration of less than 0.6–0.7 mmol/L could lead to coagulation defects. In addition, contractility of the heart and systemic vascular resistance are diminished under decreased ionized calcium levels. Because of its combined beneficial cardiovascular and coagulation effects, the calcium concentration should be monitored periodically with every ten units of transfusion, and it is recommended that a concentration of at least 0.9 mmol/L be maintained [64, 65].

### Massive transfusion protocol

Massive transfusion is typically defined as the transfusion of ten or more units of packed red blood cells within the first 24 h of injury. It is important for the resuscitation staff to identify the patients who might require massive transfusion early in the process of initial resuscitation. Following the prediction of massive transfusion, blood products should be delivered in a quick and timely manner at a high ratio of plasma, RBCs, and platelets. To achieve this quick response, not only the resuscitation staff but also the blood bank staff need to incorporate pre-implemented guidelines and flow charts for massive transfusion protocol (MTP) into their work flow [48, 66–68]. The protocol includes patient selection for activation of the MTP, description of the staff who should declare the activation, and the means by which the resuscitation team and the blood bank are informed of the protocol’s activation. In the blood bank, cooled packs of O negative RBCs, type AB FFP, and platelets will be pre-packed for quick delivery. A high-ratio pack is continually delivered each time blood is requested until the protocol is deactivated. Type-specific blood will be delivered as soon as the patient’s blood type is determined.

The MTP was implemented in 85% of the trauma centers in the USA as of 2010 [69]. The MTP is bundled with the administration of calcium, factor VIIa, and fibrinogen. The laboratory examination of coagulation function by thromboelastography is included as are other standard blood laboratory tests.

The beneficial effects of the implementation of the MTP have been reported by several authors to be reductions in mortality and in the use of blood products [67, 70, 71]. Furthermore, compliance with the protocol affects patient outcome [66]. Because it is complex to transfuse blood products in a timely and safe manner, implementation of the MTP is essential for institutions caring for severely injured trauma patients. Improved blood bank procedures, effective and efficient rewarming procedures, application of damage control techniques, and aggressive correction of coagulopathy will contribute to the survival benefit [72].

It is important to activate the MTP as quickly as possible; however, it is worth considering that massive transfusion, especially with the administration of FFP, has adverse effects for a subgroup of trauma patients. Inaba and colleagues retrospectively investigated the incidence opportunity following plasma transfusion in patients who did not require massive transfusion [73]. Although there was no improvement in survival with plasma transfusion, the overall rate of complications was significantly higher in the patients receiving plasma products.

Several scores, such as the trauma associated severe hemorrhage (TASH) [74], the scoring system developed by McLaughlin [75], assessment of blood consumption (ABC) [76], and traumatic bleeding severity score (TBSS) [77] scores, are proposed for the prediction of patients who require massive transfusion in the early phase of resuscitation. Each score includes the systolic blood pressure and heart rate on admission or after the initial resuscitation. The focused assessment with sonography for trauma exam, extremity and/or pelvic injury, sex, age, or laboratory data are assessed to calculate these scores. Recently, the TBSS score was modified to predict the need for massive transfusion more quickly [78]. The systolic blood pressure on arrival but after fluid resuscitation was used. The predictive value of the modified TBSS is still high and is reported to be equivalent to that of the TASH score.

### Remote DCR

The concept and practice of the DCR is recently applied in the pre-hospital setting and named as remote DCR (RDCR) [79]. Not only the fixed-ratio coagulation therapy using the high ratio of plasma and platelets to pRBC but also the coagulation factor concentrate-based treatment is proposed in the RDCR. It includes three major components to a step-wise approach to achieve hemostasis: (1) stop (hyper)fibrinolysis, tranexamic acid; (2) support clot formation, fibrinogen concentrate; and (3) increase thrombin generation, prothrombin complex concentrate [22]. Although RDCR warrants further investigation concerning its effect on the mortality or the blood products requirement, and the assessment of the patient’s coagulation function in the instrument limited environment, the tranexamic acid has been implemented in the RDCR in the US, French, British, and Israeli militaries as well as the British, Norwegian, and Israeli civilian ambulance services. A prospective cohort study in the civilian trauma center demonstrated reduction in mortality and multiple organ failure for patients treated with tranexamic acid in the subgroup of patients with shock [80]. In the report of Wafaisade et al., the propensity score matched analysis using the German trauma database demonstrated the prolonged time to death and reduction in early mortality in the tranexamic acid-administered trauma patients [81]. The updated European guideline suggests the administration of the first dose of tranexamic acid en route to the hospital as a grade 2C recommendation [82].

## DCR and DCS

### Adverse effects of DCS

After the recognition of the vicious cycle in trauma patients, a paradigm shift in the surgical treatment of severely hemorrhagic patients occurred. DCS was developed to challenge the lethal triad of trauma. It was originally reported by Stone and colleagues in 1983 [83] and named by Rotondo and Schwab in 1993 [20]. Since these reports, DCS has become the standard of care for the most severely injured patients. It has been widely applied not only for abdominal trauma but also for thoracic [84], vascular [85], pelvic [86], and extremity injuries [87, 88]. DCS has led to better outcomes in severely hemorrhagic patients [89]. Ten years of experience have shown that patients who receive DCS for penetrating abdominal trauma have higher survival rates and a decreased incidence of hypothermia in the operating room [90]. In the early decades after DCS was introduced, it was performed in cooperation with aggressive volume resuscitation.

Although DCS was popularized and resulted in reduced mortality, the abbreviated surgical techniques and open abdomen management led to significant increases in sub-acute complications, such as open abdomen, acute respiratory distress syndrome, intra-abdominal infections, and multiple organ failure [91]. In particular, open abdomen management resulted in an increase in severe morbidities, such as anastomotic breakdown, ventral hernias, and enteroatmospheric fistula [92, 93]. Aggressive resuscitation increased the incidence of these complications [26].

Studies have recently warned against the overuse of DCS [94, 95]. Clinical outcomes may be improved with more selective use of DCS accompanied by DCR [96].

### Changes of surgical strategy in DCR

The severely hemorrhagic patient has a limited amount of physiologic reserve before irreversible derangement, organ damage, and collapse occur. DCR restores this reserve, allowing more definitive treatment that results in decreased postoperative complications and improved outcomes [40, 68].

DCS is now incorporated as a component of DCR and should not be practiced in isolation [64]. DCR as a structured intervention should begin immediately after rapid initial assessment in the emergency room and progresses through the operating theater into the ICU in combination with DCS [48].

By starting from ground zero with the performance of DCS, DCR allows the trauma surgeon to correct the lethal triad, particularly the coagulopathy of trauma. Definitive therapy can be completed at the first operation in patients who are warm, well perfused, and without coagulopathy [97, 98].

Higa and colleagues reported that DCR increased the administration of blood products with less infusion of crystalloid solution and was associated with a survival advantage and shorter length of stay in the trauma ICU for patients with severe hemorrhage [96]. Although the number of laparotomy patients increased, the number of patients requiring damage control laparotomy decreased from 36 to 9%, and the mortality for patients requiring open laparotomy improved from 22 to 13%. The application of DCR to damage control laparotomy techniques results in an improvement in the ability to achieve primary fascia closure and decreases the requirement for staged laparotomy [99]. In addition, DCR may decrease the surgical hemostatic requirement in severely injured patients. A retrospective study showed an increase in the success rate of nonoperative management from 54 to 74% for grades IV and V severe blunt liver injury after the implementation of DCR [100]. DCR may herald the beginning of the end for DCS [98].

## Conclusions

DCR strategy is the measure that directly addresses trauma-induced coagulopathy. Although several concerns, such as the plasma to RBCs ratio, the method of achieving balanced resuscitation, and the administration of other coagulation factors, are not completely resolved, it is now the most beneficial measure for treating trauma-induced coagulopathy, and it can change the treatment strategy of trauma patients. The effect of the reversal of coagulopathy in the massively hemorrhagic patient may shift the operative strategy from one of DCS to definitive surgery.

## Abbreviations

ABC: Assessment of blood consumption
ATC: Acute traumatic coagulopathy
CRASH-2: Clinical randomization of an antifibrinolytic in significant hemorrhage 2
DCR: Damage control resuscitation
DCS: Damage control surgery
FFP: Fresh frozen plasma
ICU: Intensive care unit
J-OCTET: Japan-observational study for coagulation and thrombolysis in early trauma
MTP: Massive transfusion protocol
PROPPR: Pragmatic, randomized optimal platelet and plasma ratios
RBC: Red blood cell
RDCR: Remote damage control resuscitation
TASH: Trauma associate severe hemorrhage
TBSS: Traumatic bleeding severity score
